# Differential distribution of minerals in the proboscis hooks of *Corynosoma pseudohamanni* Zdzitowiecki, 1984 (Acanthocephala: Polymorphidae) juveniles from *Notothenia coriiceps* Richardson off Argentine Islands, West Antarctica

**DOI:** 10.2478/helm-2025-0025

**Published:** 2025-11-26

**Authors:** M. E. Caracciolo, O. M. Amin, C. Wendt, N. YU. Rubtsova, W De Souza

**Affiliations:** 1 Multiuser Center for Biomedical Phenomena Analysis (CMABio), State University of Amazonas (UEA), Manaus, Brazil; 2 Institute of Parasitic Diseases, 11445 E. Via Linda, # 2-419, Scottsdale, AZ 85259, USA; 3 lnstitute of Biomedical Sciences, Federal University of Rio de Janeiro, Rio de Janeiro 21941-590, Brazil; 4 Hertha Meyer Laboratory of Cellular Ultrastructure, Carlos Chagas Filho Institute of Biophysics, Center for Health Sciences, Federal University of Rio de Janeiro, Rio de Janeiro, Brazil

**Keywords:** *Corynosoma pseudohamnni*, West Antarctica, EDXA, localization of minerals in hooks, taxonomic implications

## Abstract

*Corynosoma pseudohamanni* Zdzitowiecki, 1984 (Polymorphidae) was described from the intestinal tract of 5 species of seals, including the type and primary host, the Weddell seal *Leptonycotes weddellii* (Lesson) in the South Shetlands, West Antarctica. *Notothenia coriiceps* Richardson was the primary paratenic host of 14 fish hosts reported in the original description. We redescribed excysted juveniles from the body cavity of *N. coriiceps* collected off Galindez Island, Argentine Islands, and included its molecular analysis, SEM images, and Energy Dispersive X-ray Analysis (EDXA) for the first time. The identity and distribution of mineral elements in the center and edge of anterior, middle, and posterior proboscis hooks establish their taxonomic relevance. Samples were dehydrated through an ascending ethanol series and then critical point dried, mounted on stubs and coated with carbon with a thickness of 20 nm. The specimens were examined and positioned using the LYRA3 FIB-SEM (TESCAN, Brno – Kohoutovice, Czech Republic), equipped with a Phoenix energy-dispersive X-ray analyzer (Oxford Instruments, Abingdon, England). X-ray spot and live scan analyses were performed at 15 kV with a spot size 2. The AZtec version 4.3 software system (Oxford Instruments, Abingdon, England) was used. We demonstrated the highest calcium levels in all hooks and hook roots compared to sulfur and phosphorus. Here, for the first time, we report a new aspect of the elemental analysis of hooks, demonstrating the differential distribution of sulfur, phosphorous, calcium, and magnesium at the center and margins of anterior, middle, and posterior hooks and hook roots. Calcium was the most prevalent element in roots (root edge and root middle) and the center of middle of all hooks. Phosphorous was equally common but not as prevalent and was highest in the middle of the roots and the middle of all hooks. Sulfur was negligible or absent in the roots and middle of all hooks but highest at hook tips and the edge of the middle hook. The biological and taxonomic importance of this EDXA pattern is discussed, as well as its relevance to hook strength and flexibility, species identity, and comparative systematics. Comparative results were also presented for cystacanths of five other species of acanthocephalans for which EDXA patterns have been studied.

## Introduction

The results of the EDXA of hooks of *Corynosoma pseudohamanni* Zdzitowiecki, 1984 (Polymorphidae) show the predominance of calcium (11.5 – 47.0 %) and, to some extent, phosphorus (2.0 – 19.6 %) in all analyzed hooks followed by lower levels of sulfur (0.0 – 7.1 %) (Amin *et al*., 2025a). Calcium levels reached the highest levels in the roots of the anterior (50.6 %), middle (67.9 %), and posterior (51.9 %) hooks. Sulfur reached higher levels at the edges of all hooks; however, we only detected the highest concentration of this element at the tip of the middle hook (7.1 %). Magnesium exhibited the lowest levels or was mostly undetectable (0.0 – 1.2 %). Magnesium was detected only in the anterior hook’s root (1.5 %). These levels of elements characterize the species of acanthocephalan being studied and evaluates the relative contribution of each element in providing strength and flexibility for attachment and retention of infection. EDXA studied cystacanths and juveniles of five other species of acanthocephalans for elemental composition. These are (1) *Southwellina hispida* (Van Cleave, 1925) Witenberg, 1932 (Polymorphidae) from the long jaw mudsucker *Gillichthys mirabilis* Cooper (Gobiidae) off the California coast (Amin et al., 2022); (2) cystacanths of *Profilicollis rancoensis* Amin, Rodríguez, Farrer, Fierro, Garcés, Rivera, D’Elía, 2023 (Polymorphidae) from the freshwater crab *Aegla abtao* Schmitt (Crustacea: Decapoda) in Ranchi Lake, North Patagonia, Chile (Amin *et al*., 2023); (3) cystacanths of *Neoandracantha peruensis* Amin, Heckmann, 2017 from the ghost crab *Ocypode guadichaudi* Milne-Edwards and Lucas in Peru (Amin and Heckmann, 2017); (4) cystacanths of *Sphaerirostris picae* (Rudolphi, 1819) Golvan, 1956 from lizards and hedgehogs in Ukraine (Amin *et al*., 2022); and (5) Cystacanths of *Moniliformis kalahariensis* Meyer, 1931 from *Blatella germanica* Linn. in India (Amin *et al*., 2022).

This study aims to characterize, for the first time, the elemental composition and spatial distribution of mineral elements—specifically calcium, phosphorus, sulfur, and magnesium—in the proboscis hooks of *Corynosoma pseudohamanni* cystacanths using Energy Dispersive X-ray Analysis (EDXA). By integrating morphological, molecular, and elemental data, we evaluate the taxonomic, biological, and structural significance of mineral localization patterns across anterior, middle, and posterior hooks and hook roots. Comparative analyses with related acanthocephalan species are included to assess the diagnostic and systematic value of hook mineralization profiles

## Materials and Methods

### Collections

A total of 594 cystacanths of *C. pseudohamanni* were collected from cysts in the body cavity of 5 individuals of the Atlantic black rockcod *Notothenia coriiceps* Richardson between November 2022 and March 2023 off Galindez Island, Argentine Islands, West Antarctica (65°15' S, 64°15' W). This collection was part of the long-term parasitological monitoring studies carried out from 2014 to 2015 by the Ukrainian Antarctic expeditions at the Ukrainian Antarctic station (UAS) “Akademik Vernadsky” on Galindez Island, West Antarctica. Acanthocephalan cysts were collected manually from fish body cavity sites, washed in saline after excystation, and kept for a few hours for proboscis evagination before fixing in 70 % ethanol. We have processed 10 specimens of each sex for SEM and energy-dispersive X-ray analysis (EDXA).

### Deposits

Voucher specimens were submitted to the Harold W. Manter Laboratory for deposition. (HWML) The collection of the University of Nebraska State Museum in Lincoln, Nebraska, USA; collection no. HWML 217883. Additional specimens are kept in the OMA collection in 70 % ethanol.

### Energy Dispersive X-ray analysis (EDXA)

EDXA is an analytical technique associated with electron microscopy used for the elemental analysis or chemical characterization of a sample. It relies on the interaction between an X-ray excitation source and the sample. Its characterization capabilities are mainly according to the fundamental principle that each element possesses a distinct atomic structure, leading to different peaks in its electromagnetic emission spectrum (Goldstein, 2003; Scimeca *et al*., 2018), which is the main principle of spectroscopy. A similar methodology to study the hooks *Moniliformis kalahariensis* Meyer, 1931 (Moniliformidae Van Cleave, 1924) was combined with TEAM (Texture and Elemental Analytical Microscopy) which is a technique that combines both crystallographic texture analysis (using Electron Backscatter Diffraction - EBSD) and elemental analysis conducted using Energy Dispersive X-ray Spectroscopy (EDS) on a single electron microscope platform (Tan, 2024). This technique allowed us to simultaneously study the microstructure and chemical composition of the *M. kalahariensis* material at the micro-scale (Amin *et al*., 2025b).

The fixed samples were dehydrated through an ascending ethanol series, which was applied, followed by critical point drying using a Tousimis Autosamdri-815 (Tousimis Research Corporation, Rockville, United States). They were mounted on stubs and coated with carbon using a sputter coater Balzers MED 010 (Balzers Union limited, Balzers, Liechtenstein.), establishing an approximate thickness of 20 nm. The specimens were examined and positioned using the LYRA3 FIB-SEM (TESCAN, Brno – Kohoutovice, Czech Republic), equipped with a Phoenix energy-dispersive X-ray analyzer (Oxford Instruments, Abingdon, England). X-ray spot analysis and live scan analysis were performed at 15 kV with a spot size of 2, and the results were recorded on charts and stored using digital imaging software attached to a computer. The AZtec version 4.3 software system (Oxford Instruments, Abingdon, England) was used. A dual-beam scanning electron microscope (SEM) equipped with a gallium (Ga) ion source was used for the LIMS (Liquid Ion Metal Source) part. The hooks of the acanthocephalans were positioned at the center of the SEM stage and sectioned longitudinally. The cut was then analyzed with X-rays at the hooks’ tip, middle, and base for chemical ions using an electron beam (FEG Schottky) to obtain an X-ray spectrum.

SEM data maps were created, marking spectra from the edge and middle of hook tips, middle, and roots. These were followed by color-coded spectra showing magnesium, calcium, phosphorous, and sulfur element overlays. Then, graphs and tables were created for each spectrum of anterior hooks (Figs. 1–11), middle hooks (Figs.12–23), and posterior hooks (Figs. 24–34).

**Figs. 1-3.**
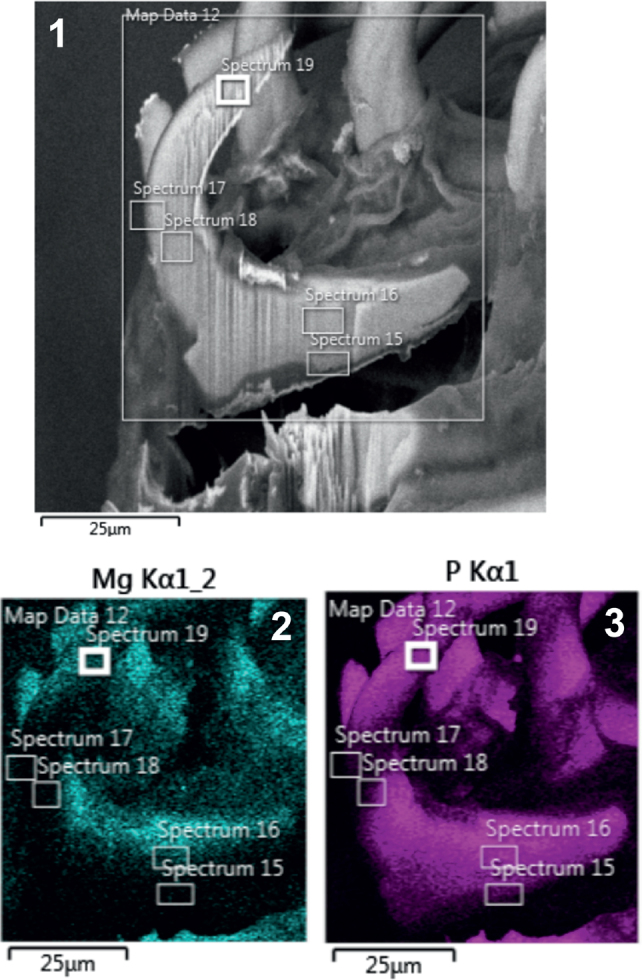
Elemental analysis of anterior proboscis hooks of *Corynosoma pseudohamanni* collected from *Notothenia coriiceps* off Galindez Island Argentine Islands West Antarctica. **1**. A Gallium-cut lateral section of an anterior hook showing spectra at which elemental analysis was performed. **2**. Phase map of magnesium. **3**. Phase map of phosphorous.

**Figs. 4-5.**
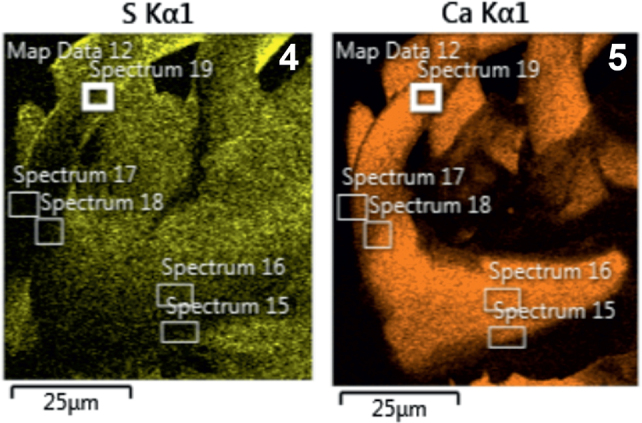
Elemental analysis of anterior proboscis hooks of *Corynosoma pseudohamanni* collected from *Notothenia coriiceps* off Galindez Island, Argentine Islands, West Antarctica. **4**. Phase map of sulfur **5**. Phase map of calcium.

**Figs. 6-7.**
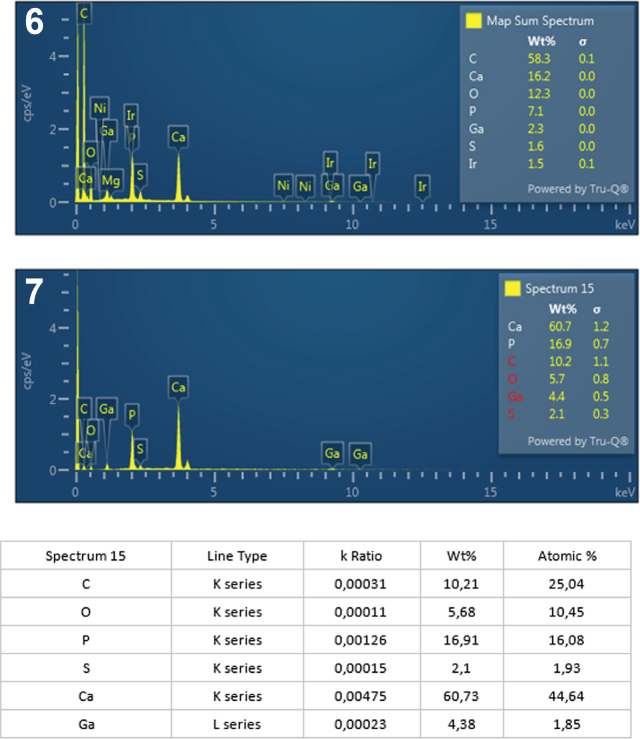
Elemental analysis of anterior proboscis hooks of *Corynosoma pseudohamanni* collected from *Notothenia coriiceps* off Galindez Island, Argentine Islands, West Antarctica. 6. Map of sum spectra with the inset showing the numerical analysis of the spectra. **7**. Graphic and tabulated levels of elements analyzed from the edge of the root (spectrum 15).

**Figs. 8-9.**
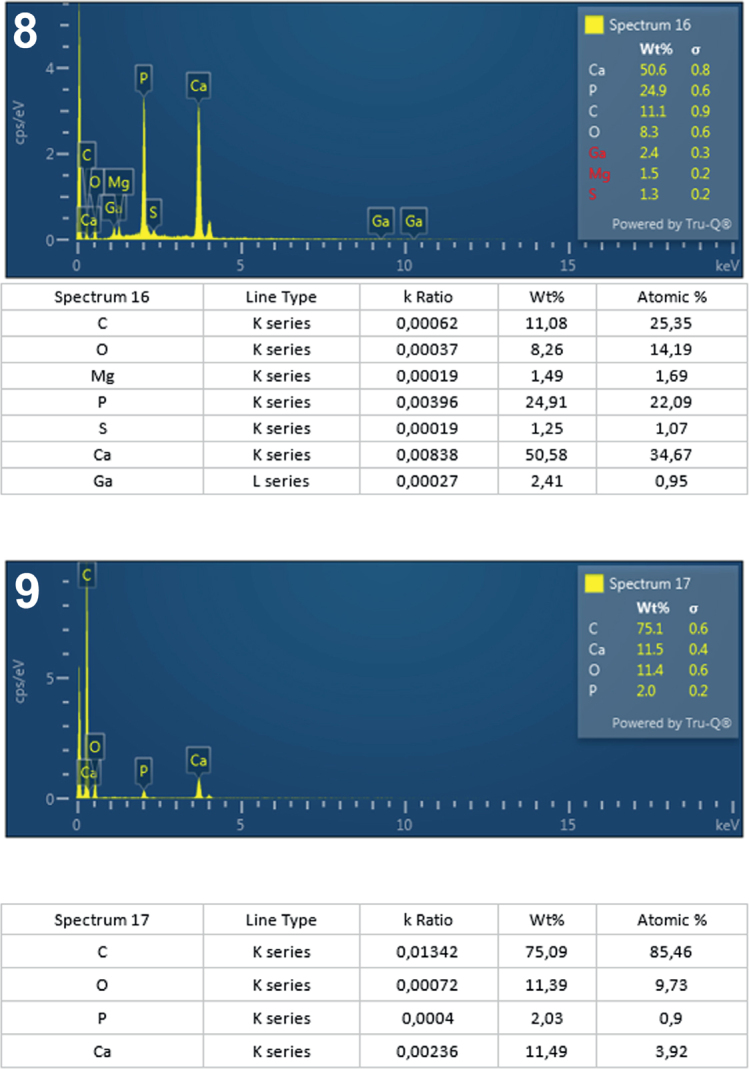
Elemental analysis of anterior proboscis hooks of *Corynosoma pseudohamanni* collected from *Notothenia coriiceps* off Galindez Island, Argentine Islands, West Antarctica. **8**. Graphic and tabulated levels of elements analyzed from the center of the root (spectrum 16). **9**. Graphic and tabulated levels of elements analyzed from the edge of the hook middle (spectrum 17).

**Figs. 10-11.**
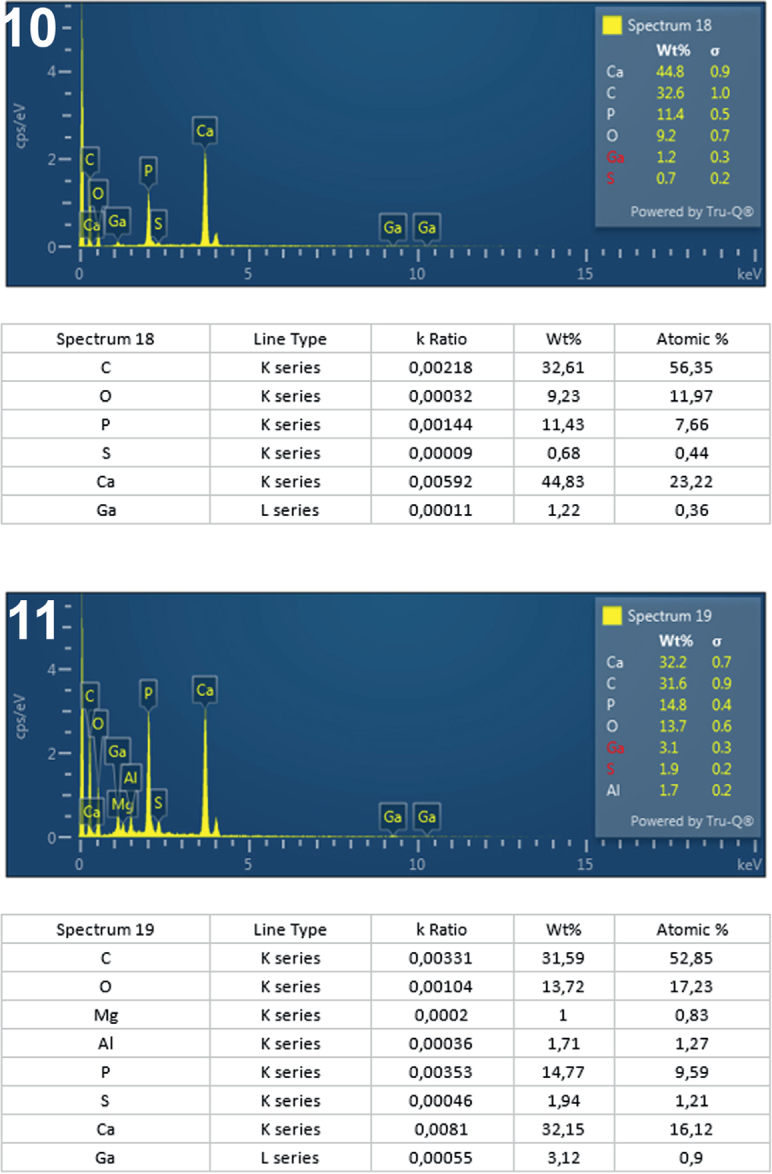
Elemental analysis of anterior proboscis hooks of *Corynosoma pseudohamanni* collected from *Notothenia coriiceps* off Galindez Island, Argentine Islands, West Antarctica. **10**. Graphic and tabulated levels of elements analyzed from the center of the hook middle (spectrum 18). **11**. Graphic and tabulated levels of elements analyzed from the hook tip (spectrum 19).

**Figs. 12-14.**
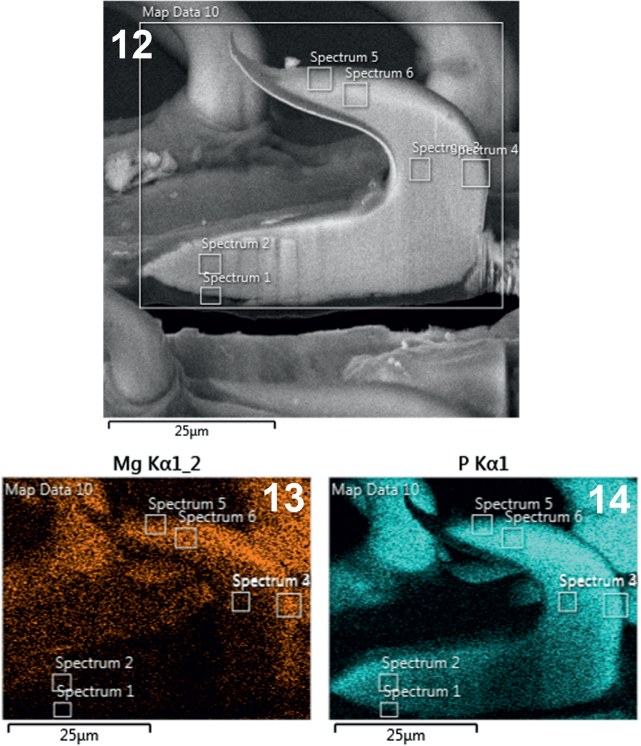
Elemental analysis of middle proboscis hooks of *Corynosoma pseudohamanni* collected from *Notothenia coriiceps* off Galindez Island, Argentine Islands, West Antarctica. **12**. A Gallium-cut lateral section of a middle hook showing spectra at which elemental analysis was performed. **13**. Phase map of magnesium. **14**. Phase map of phosphorous.

**Figs. 15-16.**
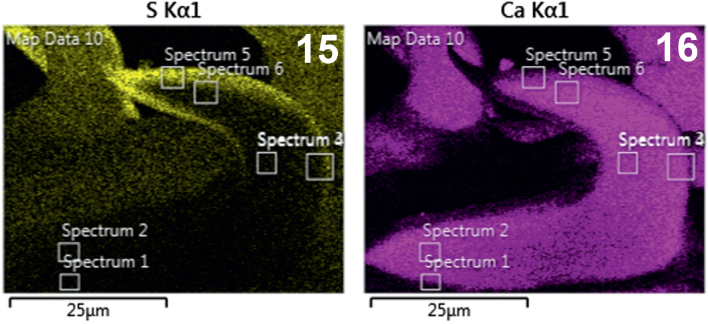
Elemental analysis of middle proboscis hooks of *Corynosoma pseudohamanni* collected from *Notothenia coriiceps* off Galindez Island, Argentine Islands, West Antarctica. **15**. Phase map of sulfur **16**. Phase map of calcium.

**Figs. 17-18.**
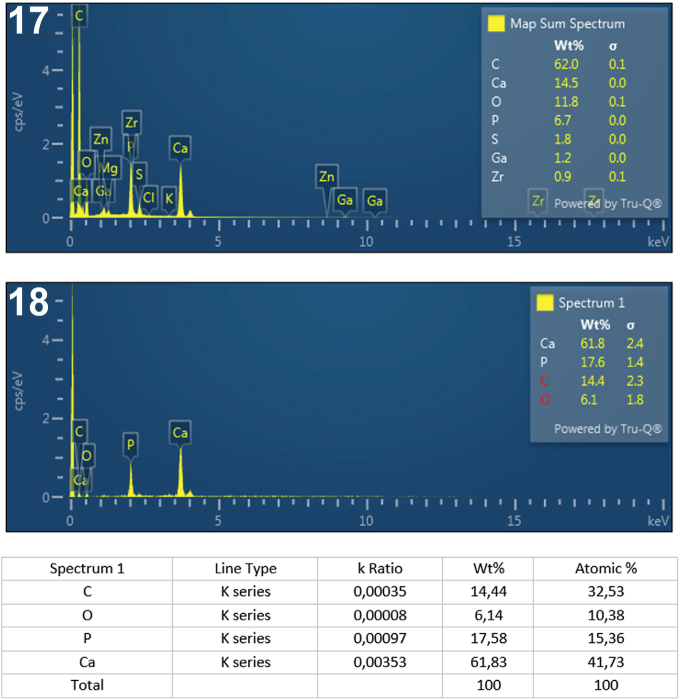
Elemental analysis of middle proboscis hooks of *Corynosoma pseudohamanni* collected from *Notothenia coriiceps* off Galindez Island, Argentine Islands, West Antarctica. **17**. Map of sum spectra with the inset showing the numerical analysis of the spectra. **18**. Graphic and tabulated levels of elements analyzed from the edge of the root (spectrum 1).

**Figs. 19-20.**
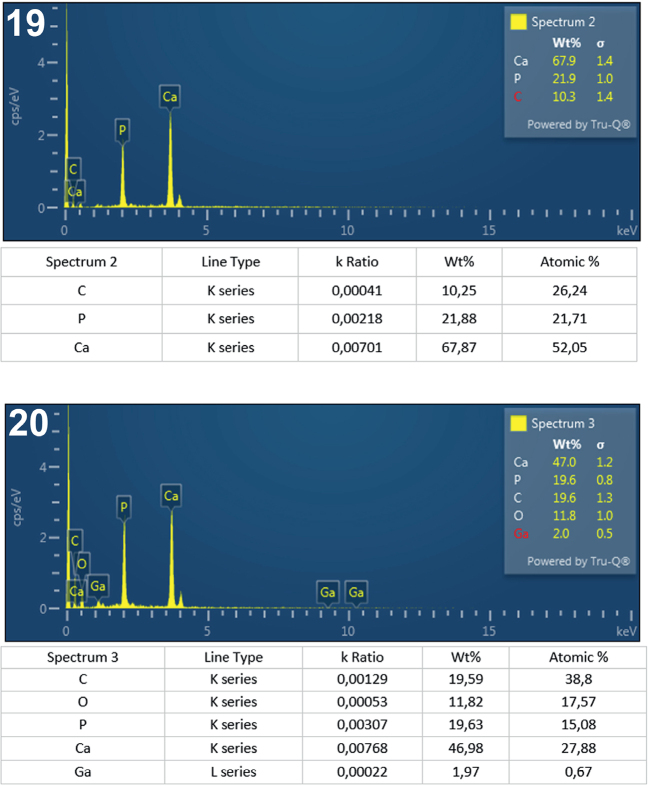
Elemental analysis of middle proboscis hooks of *Corynosoma pseudohamanni* collected from *Notothenia coriiceps* off Galindez Island, Argentine Islands, West Antarctica. **19**. Graphic and tabulated levels of elements analyzed from the center of the root (spectrum 2). **20**. Graphic and tabulated levels of elements analyzed from the center of the hook middle (spectrum 3).

**Figs. 21-22.**
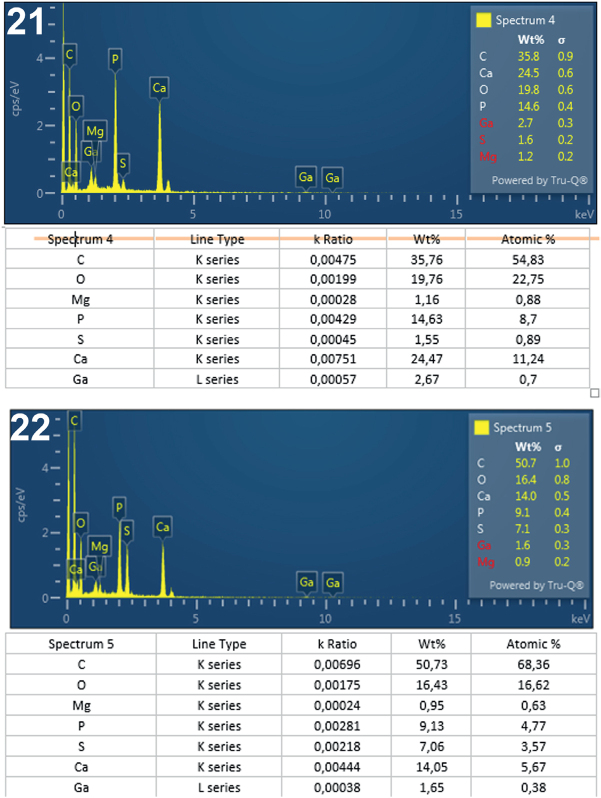
Elemental analysis of middle proboscis hooks of *Corynosoma pseudohamanni* collected from *Notothenia coriiceps* off Galindez Island, Argentine Islands, West Antarctica. **21**. Graphic and tabulated levels of elements analyzed from the edge of the hook middle (spectrum 4). **22**. Graphic and tabulated levels of elements analyzed from the edge of the hook tip (spectrum 5).

**Fig. 23.**
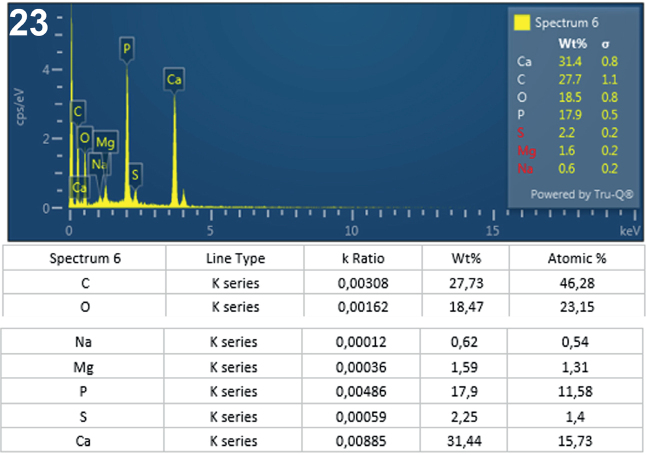
Elemental analysis of middle proboscis hooks of *Corynosoma pseudohamanni* collected from *Notothenia coriiceps* off Galindez Island, Argentine Islands, West Antarctica. **23**. Graphic and tabulated levels of elements analyzed from the center of the hook tip (spectrum 6).

**Figs. 24-27.**
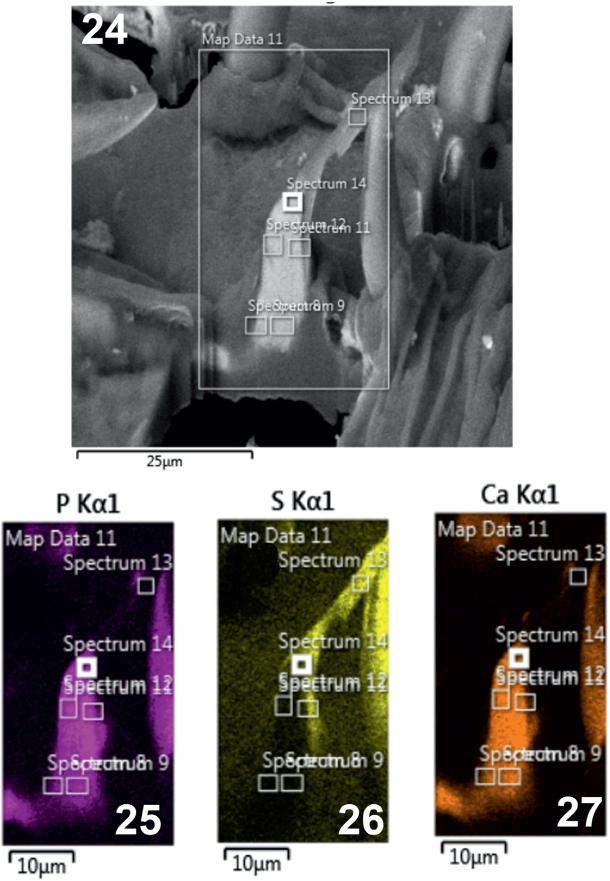
Elemental analysis of posterior proboscis hooks of *Corynosoma pseudohamanni* collected from *Notothenia coriiceps* off Galindez Island, Argentine Islands, West Antarctica. **24**. A Gallium-cut lateral section of a posterior hook showing spectra at which elemental analysis was performed. **25**. Phase map of phosphorous. **26**. Phase map of sulfur. **27**. Phase map of calcium.

**Figs. 28-29.**
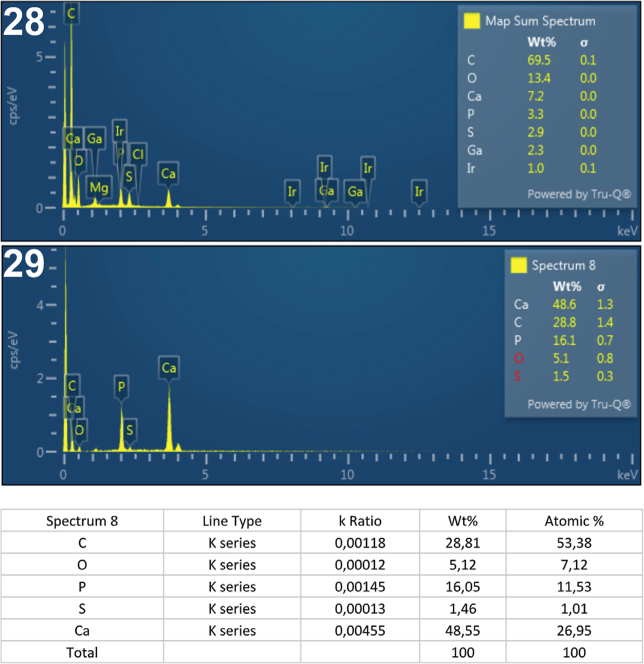
Elemental analysis of posterior proboscis hooks of *Corynosoma pseudohamanni* collected from *Notothenia coriiceps* off Galindez Island, Argentine Islands, West Antarctica. **28**. Map of sum spectra with the inset showing the numerical analysis of the spectra. **29**. Graphic and tabulated levels of elements analyzed from the edge of the root (spectrum 8).

**Figs. 30-31.**
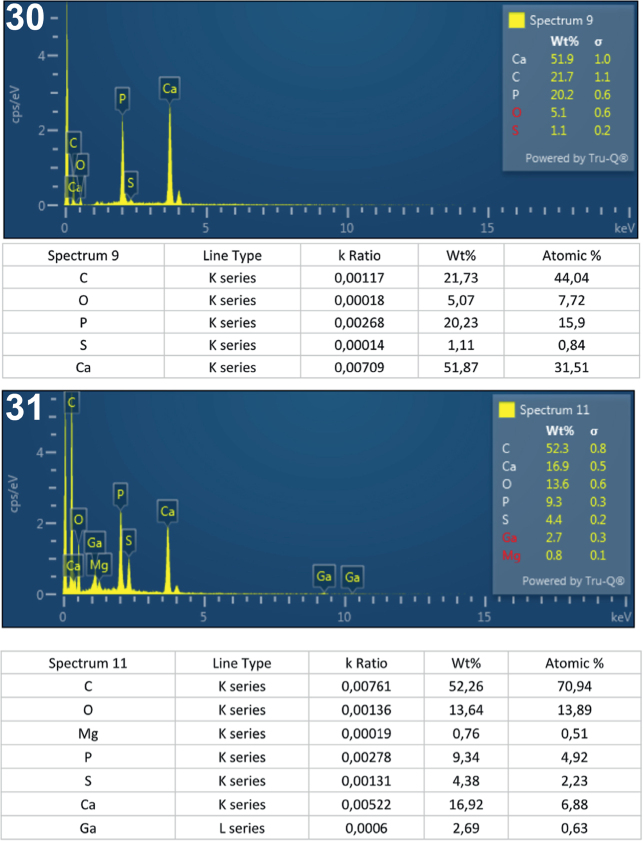
Elemental analysis of posterior proboscis hooks of *Corynosoma pseudohamanni* collected from *Notothenia coriiceps* off Galindez Island, Argentine Islands, West Antarctica. **30**. Graphic and tabulated elements analyzed from the center of the root (spectrum 9). **31**. Graphic and tabulated elements analyzed from the edge of the hook middle (spectrum 11).

**Figs. 32-33.**
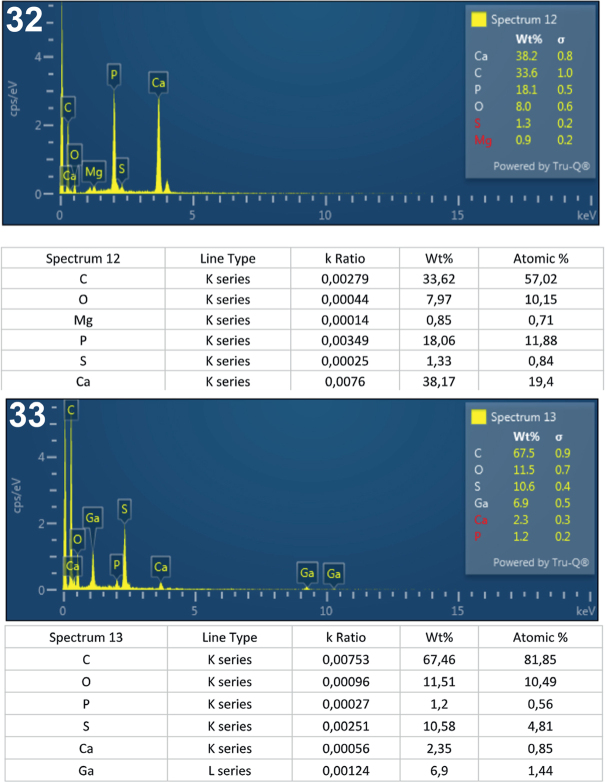
Elemental analysis of posterior proboscis hooks of *Corynosoma pseudohamanni* collected from *Notothenia coriiceps* off Galindez Island, Argentine Islands, West Antarctica. **32**. Graphic and tabulated levels of elements analyzed from the center of the hook middle (spectrum 12). **33**. Graphic and tabulated levels of elements analyzed from the hook tip (spectrum 13).

**Fig. 34.**
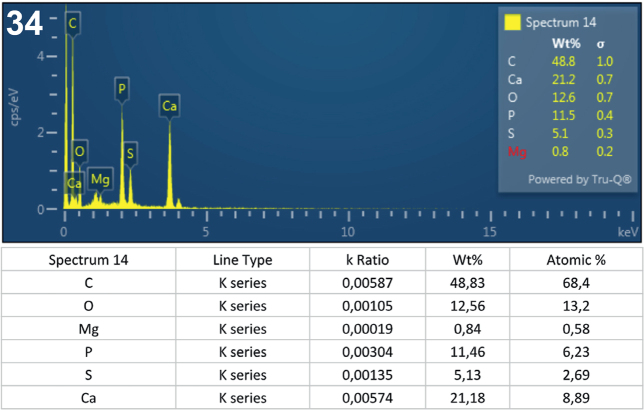
Elemental analysis of posterior proboscis hooks of Corynosoma *pseudohamanni* collected from *Notothenia coriiceps* off Galindez Island, Argentine Islands, West Antarctica. **34**. Graphic and tabulated levels of elements analyzed from below the hook tip (spectrum 14).

## Ethical Approval and/or Informed Consent

The authors declare that they have observed all applicable ethical standards.

## Results

Our results are reported as weight % for calcium, phosphorous, sulfur, and magnesium. Atomic % and K Ratios are also reported in all tables. Common elements in living cells (H, O, N) and cutting elements (Ga) are also listed in all tables and spectra but not discussed. A comparison of the levels of the primary elements (Ca, P, S) in all positions of anterior, middle and posterior hooks and roots is given in Table 1. Calcium was the most prevalent element in roots (root edge and root middle) and the middle of all hooks, (Figs. 6–11), in middle hooks (Figs. 17–23), and in posterior hooks (Figs. 30–34). Phosphorous was equally common but not as prevalent as calcium and was highest in the middle of the roots and also the middle of all hooks in anterior hooks, in middle hooks and in posterior hooks. Interestingly, phosphorous was consistent in all hook roots but was markedly lower in anterior hooks compared to middle and posterior hooks. Sulfur was negligible or absent in the roots and middle of all hooks but highest at hook tips, especially the middle hook and posterior hook as well as the edge of the middle hook. The differential distribution of these elements in certain hooks and hook regions reflects which of these attachment structures is more heavily involved in their strength and flexibility.

**Table 1.** Weight % of primary elements in different parts of hooks and hook roots of *Corynosoma pseudohamanni* collected from *Notothenia coriiceps* in West Antarctica.

Site	Anterior hooks	Middle hooks	Posterior hooks
		Calcium	
Root edge	60.73	61.83	48.55
Root center	50.58	67.87	51.87
Middle hook	11.48	46.98	16.92
Middle hook edge & center	44.83	14.05-24.47	38.17
Hook tip	32.15	31.44	2.35
		**Phosphorus**	
Root edge	16.91	17.58	16.05
Root center	24.91	21.88	20.23
Middle hook	2.03	19.63	9.34
Middle hook edge & center	11.43	9.13-14.63	18.06
Hook tip	14.77	17.90	1.20
		**Sulfur**	
Root edge	2.10	0	1.46
Root center	1.25	0	1.11
Middle hook	0	0	4.38
Middle hook edge & center	0.68	1.55-7.06	1.33
Hook tip	1.94	2.25	10.58

## Discussion

### Energy Dispersive X-ray Analysis (EDXA)

We describe the mineral distribution in the margins and center of the anterior, middle, and posterior proboscis hooks of cystacanths of *Corynosoma pseudohamanni* for the first time. We studied X-ray scans (EDXA) of FIB-sectioned hooks and spines of these acanthocephalan worms. The chemical composition of the hooks is characteristic of acanthocephalans. Hooks were evaluated for chemical ions, with sulfur (S), calcium (Ca), and phosphorus (P) being the prominent elements. Calcium and phosphorus are significant ions at the base and middle of the hooks that play a prominent role in host tissue attachment (Heckmann *et al*., 2007, 2012; Standing and Heckmann, 2014; Amin and Heckmann, 2017). Sulfur is usually high, especially at the outer edge of large hooks and hook tips. These elements play a crucial role in the mineralization of the hook, forming a hardened outer layer composed of apatite, similar to the tooth enamel of mammals. Our results support these findings and are close to those of the closely related *Corynosoma paraevae* Amin, Chaudhary, Caracciola, Rubtsova, Wendt, Lisitsyna, Kuzmina, de Souza, Singh 2025 collected from *N. coriiceps* in Galindez Island, West Antarctica with 0 – 4.15 % S, 15.11 – 50.55 % Ca, 6.55 – 23.69 % P and 0 – 1.87 % Mg (Amin *et al*., 2025c). Magnesium probably plays a role in the mineralization of hooks, similar to the disulfide bonds formed by sulfur in the protein apatite (Amin & Heckmann, 2017). Sodium, a rarely prominent metal, was found in scans of whole hooks of some other acanthocephalans.

EDXA, as a diagnostic tool, supports the observation that populations of an acanthocephalan species will consistently have similar EDXA spectra irrespective of host species or geography. The taxonomic identity of species is deep-seated at the genetic level, manifesting the organism’s morphology and biochemistry as revealed, in part, by its elemental spectra (Amin *et al*., 2022).

The results of large and small gallium (Ga) cut hooks are given in Figs. 6–11, 17–23 and 28–34. The elements necessary for the mineralization and hardening of the hooks, especially calcium and phosphorus, are present with sulfur in minimum amounts. In contrast, the percent of sulfur was highest in hook tips and hook edges of *Cavisoma magnum* (Southwell, 1927) Van Cleave, 1931 from *Mugil cephalus* in the Arabian Sea, reaching 43.51 wt. % and 27.46 wt. %, respectively (Amin *et al*., 2018).

Metal analysis of hooks has become a diagnostic standard since hooks have the highest level of elements compared to the mid-and posterior trunk regions of the acanthocephalan body (Heckmann *et al*., 2012). Specifically, the sulfur content in the proboscis is paramount in the composition of disulfide bonds in the thiol groups for cysteine and cystine of the polymerized protein molecules. In *C. pseudohamanni*, sulfur levels were highest at 10.58 % at the tip of posterior hooks (Table 1). Protein synthesis occurs in transcription and translation (Stegman, 2005). The formed disulfide bonds are direct by-products of the DNA-based protein synthesis process, which makes up a biological species’ identity. Accordingly, the level of sulfur in our EDXA profiles will indicate the number of sulfur bonds that, along with the levels of calcium phosphates, will characterize the identity of a species based on its nuclear DNA personality (Amin *et al*., 2022). Diversity in the species-specific EDXA profiles will further substantiate its taxonomic relevance in the cystacanth and adult stages when such information becomes available.
